# Clinical analysis of bronchoscope diagnosis and treatment for airway foreign body removal in pediatric patients

**DOI:** 10.1186/s13052-022-01347-x

**Published:** 2022-09-02

**Authors:** Lin-Lin Han, Chen Meng, Zhong-Xiao Zhang, Xiao-Di Tang, Jing Ma, Chang-Xiao Li

**Affiliations:** Center for Respiratory Intervention, Children’s Hospital Affiliated to Shandong University, Jinan, China

**Keywords:** Airway foreign body, Child, Bronchoscope, Suffocation

## Abstract

**Background:**

Along with the wide spread application and technical development of the flexible and rigid bronchoscopy, the airway foreign body removing method cme to the specific technique for different foreign bodies from the single foreign body forceps.

**Methods:**

Selected 633 children who were diagnosed as airway foreign bodies by the Department of Respiratory Intervention, Children's Hospital affiliated to Shandong University from January 1st, 2018 to December 31st, 2021, and the airway foreign bodies were diagnosed using bronchoscopy. After comprehensive assessment of the foreign body nature in the airway, the foreign bodies were removed by freezing, laser, electrocoagulation, balloon and other techniques, the success rate of the foreign body removed from the airway was observed, the percentage of the foreign body removed using different techniques, the operation time, and the incidence of post-adverse reactions during operation.

**Results:**

The success rate using flexible bronchoscope alone to remove foreign bodies in the airway was 99.2%. After flexible bronchoscopy, 19 cases of foreign bodies were removed by vacuum suction alone, 513 cases were removed by foreign body forceps alone, 62 cases were combined with cryotherapy, 2 cases were electrocoagulation, 6 cases were mesh baskets, 3 cases were balloons, 5 cases were laser, and various 18 cases of foreign bodies were invloved by technical combination. 5 cases of flexible bronchoscope combined with rigid bronchoscope combined to remove foreign bodies. The operation time was from 5 min to 1 h, with an average of 20 min. There were 17 cases of hypoxemia (2.7%) during operation, 36 cases (5.7%) of bleeding caused by airway mucosa injury after treatment, and 70 cases (11.2%) of laryngeal edema. The total incidence of adverse reactions was 19.6%, there were no deaths due to foreign bodies and treatment.

**Conclusions:**

According to different properties of airway foreign bodies, it is safe and effective to select appropriate techniques to remove foreign bodies using the flexible bronchoscope, which can increase the removal rate of airway foreign bodies and reduce the occurrence of serious complications.

## Background

Foreign body (FB) refers to FB in the larynx, trachea and bronchi, which is more frequent in infants and children less than 3 years old, upper respiratory tract obstruction can occur if treatment is not on time, and it can endanger life of the child in severe cases [1]. Early and accurate diagnosis and early removal of airway FB can significantly reduce airway complications. With the wide spread application and technological development of flexible bronchoscopes, various effective methods were adopted for different FB using flexible bronchoscopes. In the past 3 years, we have used flexible bronchoscopes, combining with foreign body forceps, mesh basket, cryosurgery, laser and balloon etc., successfully removed FB in the airway of 628 children, and reported as follows.

## Materials and methods

### Subject

From January 1st, 2018 to December 31st, 2021, 633 cases of children with airway FB were considered on chest computerized tomography (CT) and clearly diagnosed by bronchoscopy in the Department of Respiratory Intervention, Children's Hospital affiliated to Shandong University, including 448 males and 185 females; age from 6 months ~ 14 years old (average age is about 1.8 year).

### Method

#### Examination and treatment equipment

(1) Electronic bronchoscope: Evislucer ABF-260 series of electronic bronchoscope produced by Opus Company of Japan: BF-P260F (outer diameter 4.0 mm, working channel 2.0 mm; Bf-xp260f (outer diameter 2.8 mm, the working channel 1.2 mm). (2) Laser treatment equipment. (3) Electrocoagulation equipment: Including German ERBE VI0200D + APC2 respiratory endoscopic electro surgery workstation system, OlympusCD-6C-1 column electrocoagulation head. (4) Cryotherapy equipment: freezer: produced by Beijing Kulan Company, the outer diameter of the freezing probe is 1.8 mm. Refrigerant: carbon dioxide. (5) Balloon: according to the position of the foreign body mound and the different range of the balloon that can be entered, the balloon of BostonScientific Company, Medtronic balloon and 5061 high-pressure gun pump were selected respectively**.**

#### Preoperative preparation

Blood routine, coagulation routine, infectious markers, electrocardiogram, cardiac ultrasound and chest CT scan were performed before examination and treatment (enhanced scan + 3D airway reconstruction for children with relatively long onset time). Food and water were forbidden for more than 6 h before examination to prevent aspiration caused by intraoperative vomiting.

#### Procedure

- (1) Under general anesthesia, the bronchoscope enter through the nasal cavity, spray diluted lidocaine through the treatment channel after passing through the glottis for total airway surface anesthesia, remove and aspirate airway secretions, enter the healthy side according to the imaging examination conclusions, and then check each lobe, segment, and sub-segment bronchus on the affected side.
- (2) Once the foreign body was found in the airway, the nature, size, shape, and location of the foreign body will be detected. Based on the appropriate angle of the foreign body forceps, gently pull the foreign body with clamping the foreign body, which can indicate that the foreign body is obviously incarcerated in the lumen or not. Small non-sharp FB can be attached to the head of the bronchus by vacuum suction, the foreign body forceps and bronchoscope can be withdrawn slowly together. If the foreign body is incarcerated or there is a small amount of granulation tissue wrapped making it diffcuilty to remove, in this case the foreign body should be clamped using foreign body forceps and loosen from different directions. If there is more granulation tissue surrounding the foreign body, use freezing or electrocoagulation to clean up the granulation tissue, and give active local hemostatic treatment if there is bleeding. After fully exposing the foreign body, select the foreign body forceps or mesh basket based on the shape, size and location of the foreign body.
- (3) After removing the foreign body, it is treated with freezing according to the local airway bleeding, granulation hyperplasia and mucosal damage. After operation, the bronchoscope should be reviewed in a timely manner, and necrotic materials should be cleaned up, and granulation tissue frozen or electro coagulated if necessary.

### Clinical observation index

The FB removal success rate, the percentage using different techniques, operation time, and the incidence of adverse reactions were recorded among these 633 cases.

## Results

### Types of foreign body

**Fig. 1:**
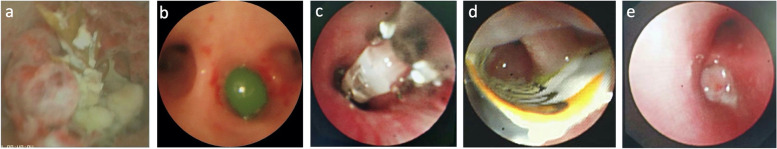
The appearance of foreign bodies lodged in the bronchus after bronchoscopy. **a** Pine needle foreign body, surrounded by granulation tissue wrapped, and adhesion of serious infection. **b** Green gelatinous round foreign body with smooth surface. **c** LED wick foreign body, the surface is sharp. **d** The beginning of the candy paper foreign body is sharp, and the surrounding granulation is wrapped. **e** The blood supply of granulation tissue wrapped on the surface of unknown foreign body is rich

Among the 633 cases children with airway FB, 564 cases were plant FB, including peanuts (shells), melon seeds (shells), walnut kernels, chestnuts (shells), soybeans, pulp (shells, stems), scallions, pine nuts, almonds, etc., 7 cases of plastic products, 5 cases of milk tea beads, 8 cases of pen caps, 1 case of the wick for LED lamp, 25 cases of bone-like FBs, 2 cases of grass spikes, 2 cases of pine needles, 1 case of spicy striped meat, 2 cases of teeth, 2 cases of hair, particles there were 2 cases of unknown objects, 1 case of thread ends, 2 cases of packaging bags, and the remaining 9 cases were all kinds of unknown foreign objects (Fig. 1) (Table 1). The above airway FBs were removed in 628 cases at one time, and the success rate was 99.2% using flexible bronchoscope alone. The remaining 0.8% of AIRWAY FBs were removed with flexible bronchoscopy combined with rigid bronchoscopy.

**Table 1:** Type of foreign body

FAB	N	%
Plant FBs	564	89.10%
peanut (or shell)	99	15.64%
melon seed (or shell)	68	10.74%
walnut kernel	56	8.85%
chestnut (or shell)	72	11.37%
soybean	45	7.11%
pulp (or shell, or stem)	79	12.48%
scallion	59	9.32%
pine nut	53	8.37%
almond	33	5.21%
plastic product	7	1.11%
milk tea bead	5	0.79%
pen cap	8	1.26%
the wick for LED lamp	1	0.16%
bone-like FB	25	3.95%
grass spike	2	0.32%
pine needle	2	0.32%
spicy striped meat	1	0.16%
teeth	2	0.32%
hair	2	0.32%
particle (unknown object)	2	0.32%
thread ends	1	0.16%
packaging bag	2	0.32%
unknown object	9	1.42%

### Clinical features

Among the 633 cases with clinical manifestations, 602 cases (95.1%) of cough, 393 cases (62.1%) of wheezing, 252 cases (39.8%) of causing, 328 cases (51.8%) of repetitive respiratory infection, 11 cases (62.73%) of hemoptysis; 567 cases (89.6%) of asymmetric breath sounds, 482 cases (76.1%) of audible stridor; 467 cases (75.2%) of with a history of foreign body inhalation, 399 cases (63.0%) of concurrent pneumonia, 538 cases (85%) of emphysema, 146 cases (23.1%) of atelectasis and 152 cases (24.0%) of bronchitis.

### Foreign body removal technique and duration

**Fig. 2:**
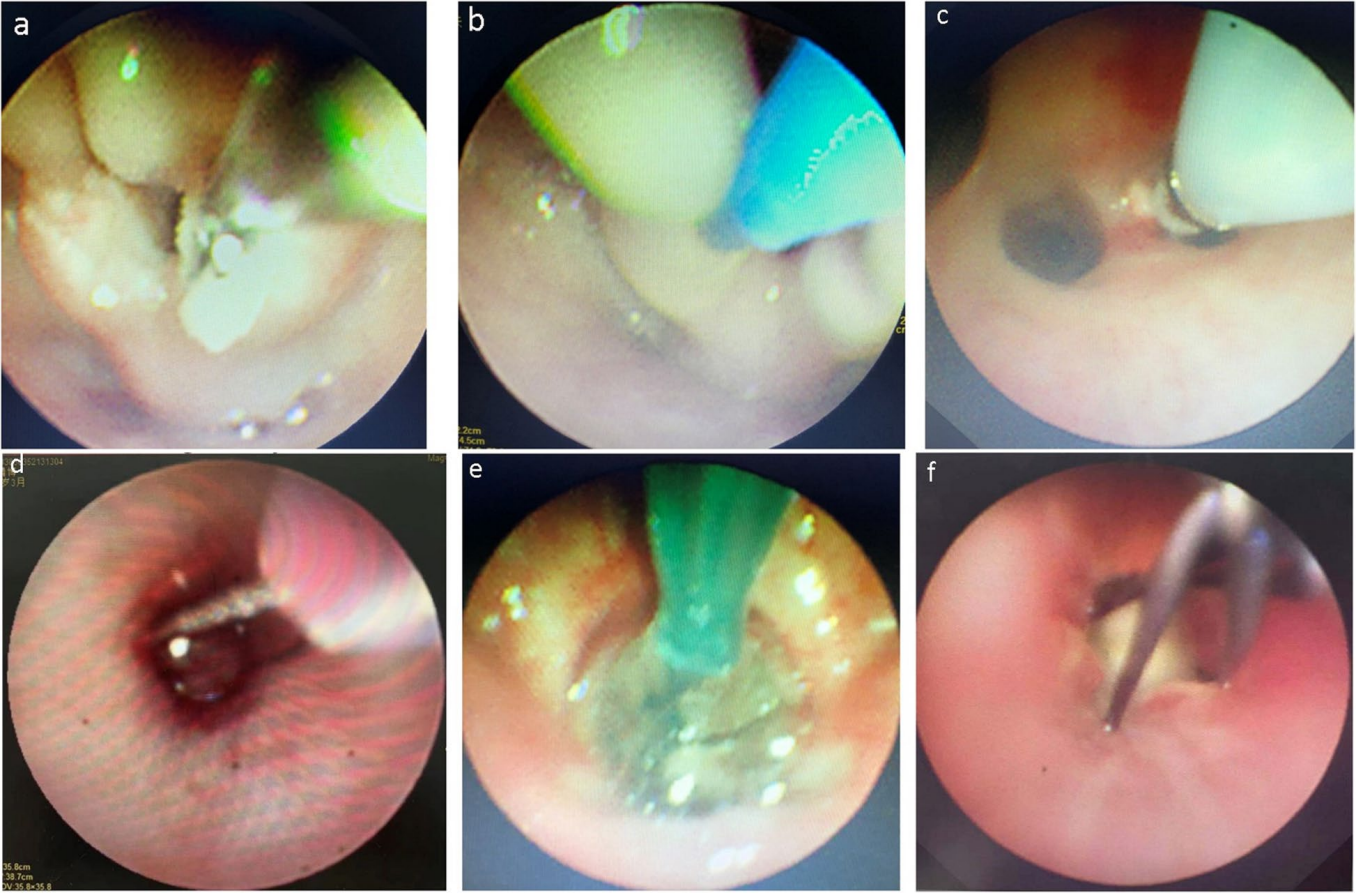
The treatment of bronchial foreign body under bronchoscopy was performed with many techniques. **a**.Trachea foreign body forceps was used to remove airway foreign body. **b** The granulation tissue surrounding the foreign body is being treated with laser. **c** Granulation tissue stimulated by bronchial foreign body was treated with cryotherapy after removal of bronchial foreign body. **d** The granulation tissue surrounding with abundant blood supply of the foreign body was treated with electrocoagulation. **e** After the balloon dilation technique was applied to the distal end of the airway where the foreign body was, the foreign body was pulled to the proximal end of the airway and removed. **f** Mesh basket was used to get the foreign body from the airway

Negative pressure suction and removal of FB in 19 cases, FB forceps alone in 513 cases, FB forceps combined with freezing in 62 cases, electrocoagulation in 2 cases, mesh baskets in 6 cases, balloons in 3 cases, lasers in 5 cases, and multiple techniques for combined removal of FB in 18 cases (Fig. 2). Five cases were combined with rigid bronchoscope to remove FB (Table 2). They were one walnut kernel, two pen caps, one plastic foreign body, and one stone-like FB. The removal of the above FBs takes about 5 min-1 h, and the average is 20 min.

**Table 2:** Foreign body removal technique

	N	%
Negative pressure suction	19	3.00%
FB forceps (A)	513	81.04%
A with		
Freezing (B)	62	9.79%
Electrocoagulation (C)	2	0.32%
Mesh basket (D)	6	0.95%
Balloon (E)	3	0.47%
Laser (F)	5	0.79%
Multiple techniques combined	18	2.84%
A + B + E	5	0.79%
A + B + D	7	1.11%
A + B + D + E	6	0.95%
With rigid bronchoscope	5	0.79%

### Complications and prognosis

There were totally 123 cases (19.6%) of complications which occurred during removing FB under flexible bronchoscopy, mainly 17 cases (2.7%) of hypoxemia during bronchoscopy, which were relieved by increasing the oxygen flow and suspending the operation during the operation; 36 cases (5.7%) of bleeding caused by airway mucosal injury were treated with local adrenaline and thrombin, and if necessary, intravenous hemostatic drugs which is pituitrin was given and all were improved. No operation related massive hemoptysis was observed; 70 cases (11.2%) there was mild laryngeal edema, which improved after treatment with adrenaline and corticosteroids. No postoperative dyspnea was observed. There was no other serious complications such as postoperative pneumothorax and mediastinal emphysema, and no deaths.

## Discussion

Airway FB is the most common respiratory emergency in children. The foreign body aspiration in the airway is usually divided into three stages [2]. The first stage is the sudden impact of the FB into the airway, leading to acute cough, stridor, respiratory distress and cyanosis. This period is the most dangerous. If the foreign body is trapped in the glottis and airway, it may cause suffocation and even death. The patient usually progresses to an asymptomatic stage, followed by airway FBs that are trapped in a fixed position in the tracheobronchial tree, and the airway reflex gradually weakens over time. The third stage involves complications secondary to chronic airway FB, manifested as infections, such as recurrent pneumonia, chronic cough, unilateral wheezing, or asthma-like symptoms. Further delay in the diagnosis of airway FB may lead to bronchiectasis and permanent damage to lung tissue. Therefore, the children with recurrent cough, wheezing, hemoptysis, lung inflammation and other unknown causes after repeated anti-inflammatory treatments should be highly vigilant for airway FB, and bronchoscopy should be performed as soon as possible to confirm the diagnosis and treatment.

Studies reported that rigid bronchoscopy was used as the gold standard for diagnosis and treatment of FB in the airway [3]. The rigid bronchoscopy can maintain effective mechanical ventilation through the breathing circuit without retaining general anesthesia, while exploring the airway to determine the location of foreign bodies, and can enter the bronchial segment of the leaf segment, so that the airway forms a straight channel, which is convenient for rigid bronchoscopy. The foreign body forceps linear forceps can improve the success rate of foreign body removal, especially for foreign bodies with irregular shapes such as bones, pen caps, peanuts, etc., with large volume and incarceration resistance. In this study, 2 cases with pen caps, 1 case with walnut kernels and 1 case with plastic foreign bodies were foreign bodies with large volume and incarceration resistance, so the flexible bronchoscope combined with the rigid endoscope was required to be used together. Another case of fruit core-like foreign body was incarcerated in the subglottic airway, and the location was special, and it was difficult to remove with the rigid endoscope or the flexible bronchoscope alone. Because the foreign body is too large to damage the glottis, it also avoids the residual foreign body in the distal airway. Since the rigid bronchoscope can be fixed at a certain position in the bronchus to form a straight airway, the inner diameter of the segmental bronchus in children under 2 years old is about 2–5 mm, but with the deepening of the location of the foreign body, especially the foreign body in the bronchus below the lung segment, rigid bronchial The scope can only be selected with 3.0 inner diameter (inner diameter 4.3 mm, outer diameter 5.0 mm), narrow tube,smallvisual range, there is the blindness during clamping, so it is easy to push the foreign body into the deeper position or bite the bronchus by wrong forceps. Ridge leading to local oozing. In recent years, the flexible bronchoscopy has been widely used in clinical operation, as it is more safe. With skilled operation by physicians, the flexible bronchoscopy can be conducted under local anesthesia and sedation. Will repeatedly rub the vocal cords to avoid severe edema in the glottis. A small amount of sedation can be tolerated well, and the bronchi or upper lobe bronchi, including subsegmental bronchi, that are not easily visible on rigid bronchoscopy can be clearly seen, usually up to grade 6 or 7. The rigid bronchoscope is limited by the equipment and cannot be used for patients with cervical spine, mandible or head abnormalities, because the neck is not suitable for extension and mandibular fixation; it is also not suitable for foreign bodies in the peripheral airways, especially those in the upper lungs. At the same time, the operation of rigid bronchoscopy is more difficulty, and the qualified personnel is limited, which also impact the application. Furthermore, the flexible bronchoscope can locally flush the inflammatory site during the process of clamping the bronchial foreign body, and if necessary, can remove the abnormally proliferated granulation tissue, thereby helping the absorption, discharge and recovery of local inflammatory exudates. The airway is unobstructed, the disease duration was shortened, and the complications was reduced. Due to the continuous stimulation of the bronchi by exogenous bronchial foreign bodies, the bronchial mucosa is prone to granulation tissue hyperplasia, which can further lead to complications such as atelectasis, bronchiectasis, pneumonia, etc., such as secondary bronchiectasis, which are often local, and non-full lung diffuse, so after flexible bronchoscope treatment, foreign body stimulation can be removed as soon as possible, which may reduce complications, reduce further invasive treatment operations, and prevent surgical treatment maximum. In this study, the complication rate during and after flexible bronchoscopy in children with airway foreign bodies was 19.6%, and there were no deaths due to FB and treatment. This is consistent with many reports in the literature [4–6]. Therefore, flexible bronchoscopy has become a commonly used method for removing FB in the airway of children.

In this study, the airway FB removal with the flexible bronchoscope were mainly edible plant FB, which may be related to the immature swallowing mechanism of the child [7]. The success rate of the flexible bronchoscope for removing FB was different in the studies Tang et al. used flexible bronchoscope to remove FB from the airway of 1027 children with a success rate of 91.3% [8]. Rodrigues et al. reported that the success rate of using 33 cases of flexible bronchoscope to remove FB was 82.5% [9]. In this study, 628 cases of FBs were removed with the flexible bronchoscope, and the success rate of removal was 99.2%. Under normal circumstances, the flexible bronchoscope treatment port can smoothly grasp a part of the airway FB through the foreign body forceps, and some FBs due to the abnormal size, texture, and shape, or due to the long period in the airway, the surrounding granulation tissue proliferation is obvious or even the FB is completely wrapped, and it is difficult to remove the foreign body smoothly with foreign body forceps. For the removal of airway FB of different natures, the flexible bronchoscope can be used alone or in combination with foreign body forceps, net baskets, freezing, balloons, lasers and other technologies. For a small number of intractable airway FB, when there is a risk of massive hemoptysis, airway perforation, etc., surgical intervention is ultimately required [10, 11]. The 628 cases of airway FBs in this group were successfully removed except 5 cases with rigid bronchoscopy and 123 cases had mild side effects, which were all improved after treatment. There were no major hemoptysis, dyspnea, pneumothorax and other conditions caused. The experience is summarized as follows: (1) For FB that have the short retention time and are particularly small, non-sharp FB can be directly adsorbed on the end of the bronchial lens by negative pressure and slowly moved out of the airway with the bronchoscope. (2) FB with irregular shapes, relatively rough surfaces, and movable FB can be fine-tuned to the appropriate gripping position with foreign body forceps. The clamps are slowly moved out of the airway with the bronchoscope. (3) Smooth, spherical or elliptical, fragile and movable FB. The foreign body can be taken out by freezing or mesh basket. (4) The incarcerated foreign body can be taken out by forceps, and the balloon will slowly pass through the gap between the airway and the foreign body, enter the distal end of the foreign body, expand and pull and cause the foreign body to loosen. Use forceps or mesh basket to take out the remaining FB (5) The foreign body that is wrapped and covered by granulation tissue can be removed by laser, freezing or electrocoagulation to clean the surrounding granulation tissue, and then the foreign body clamp or mesh basket is used to take out the foreign body.

## Conclusions

In summary, for those who have repeated coughing, wheezing, and recurrent respiratory symptoms after anti-inflammatory treatment, the foreign body is highly suspected, and the bronchoscopy should be performed as soon as possible. For airway foreign bodies of different natures, the bronchoscopy should be combined with appropriate bronchoscopy based on the type, location, size, firmness of incarceration, and granulation encapsulation, which can not only improve the removal rate of airway foreign bodies, but also can shorten the operation duration and reduce the occurrence of complications.

## Data Availability

The datasets used and/or analyzed during the current study are available from the corresponding author on reasonable request.

## Abbreviations

FB: Foreign body
CT: Computerized tomography
